# Strategies for laboratory professionals to drive laboratory stewardship

**DOI:** 10.1016/j.plabm.2021.e00249

**Published:** 2021-07-24

**Authors:** Terra E. White, Wesley B. Wong, Diane Janowiak, Lee H. Hilborne

**Affiliations:** a Quest Diagnostics, 500 Plaza Dr, Secaucus, NJ, 07094, USA; b Hc1, 6100 Technology Center Dr, Indianapolis, IN, 46278, USA; c Department of Pathology and Laboratory Medicine, University of California, Los Angeles, 757 UCLA Medical Plaza, Room B788, Los Angeles, CA, 90095, USA

**Keywords:** Clinical laboratory, Laboratory stewardship, Quality of health care, Change management, Patient care, Leadership

## Abstract

Appropriate laboratory testing is critical in today's healthcare environment that aims to improve patient care while reducing cost. In recent years, laboratory stewardship has emerged as a strategy for assuring quality in laboratory medicine with the goal of providing the right test, for the right patient, at the right time. Implementing a laboratory stewardship program now presents a valuable opportunity for laboratory professionals to exercise leadership within health systems and to drive change toward realizing aims in healthcare. The proposed framework for program implementation includes 5 key elements: 1) a clear vision and organizational alignment; 2) appropriate skills for program execution and management; 3) resources to support the program; 4) incentives to motivate participation; and, 5) a plan of action that articulates program objectives and metrics. This framework builds upon principles of change management, with emphasis on engagement with clinical and administrative stakeholders and the use of clinical data as the basis for change. These strategies enable laboratory professionals to cultivate organizational support for improving laboratory use and take a leading role in providing high-quality patient care.

Establishing the correct diagnosis is fundamental to the appropriate treatment of patients, making laboratory medicine and other diagnostic services (eg, radiology) critical to the overall practice of medicine. Some suggest that up to 70 % of medical decisions are based, in part, on laboratory findings [1]. While the specific percentage may be debatable, there is no question that laboratory diagnostics are central for medical practice today. The coronavirus disease 2019 (COVID-19) pandemic highlighted the critical role that the nation's laboratories and laboratory professionals play in assuring quality healthcare, well beyond just the pandemic response. Healthcare leaders repeatedly acknowledged the importance of laboratory testing for COVID-19 patient management to, for example, assess symptomatic patients, identify emerging variants, and perform effective contact tracing [2]. Laboratory medicine is also central to the worlds' public health response to COVID-19 [3].

Laboratory testing is typically one of the highest volume medical activities in a health system [4]. Despite their major role in driving clinical decision-making, laboratory diagnostics are highly variable and filled with opportunities to increase appropriate testing while reducing waste. Annual cost estimates in the US for low-value screening, testing, or procedures range from $17.2 billion to $27.9 billion [5]. Excessive diagnostic testing can also increase patient risk while not improving diagnostic certainty [6]. Achieving the aims of higher quality and lower cost depends on the laboratory community taking a central role in improving effective test utilization.

Over the last century, pathologists and laboratory professionals honed their craft while simultaneously expanding tools available to screen, diagnose, treat, and manage patients. The laboratorian historically focused quality efforts on a test's analytic quality. While analytic quality is necessary for appropriate care, it is not sufficient; effective test utilization requires clear medical justification to support the need for a particular test with an understanding of how the results will contribute to guiding patient management. This discussion focuses on the evolution of effective test utilization and implementation of laboratory stewardship as a laboratory leadership strategy to assure effective test use.

## 1 Evolution of quality in laboratory medicine

The pursuit of laboratory medicine quality improvement will shortly celebrate its 100th anniversary. In 1922 a group of 39 physicians came together to form what is now known as the American Society for Clinical Pathology (ASCP), now the largest professional organization of pathologists and laboratory professionals in the world [7]. The goal of the ASCP was to “achieve greater scientific proficiency in clinical pathology [meaning here the practice of pathology in the clinical setting, including both anatomic and clinical pathology], and to maintain the status of clinical pathologists on an equal plane with other specialists.” [7] ASCP recognized the contributions and importance of both quality people and quality processes to assuring reliable laboratory results. As such, ASCP was instrumental in creating the American Board of Pathology (1935) to certify pathologists and the Board of Registry (now the Board of Certification) to certify laboratory professionals (1928) [7]. ASCP, under the guidance of past president F. William Sunderman, created an interlaboratory quality control program, now recognized as the College of American Pathologists’ Proficiency Testing Program [8,9].

The evolution of quality in pathology and laboratory medicine paralleled that of medicine generally. In 1910 Abraham Flexner issued his landmark report that fundamentally restructured medical education in the United States, a structure still used today. Training moved from a relatively unstructured generalist model to focus on scientific methods with considerably greater rigor [10]. All medical disciplines reexamined their roles in medical education, delivery of healthcare, and patient centricity; pathology and laboratory medicine was no exception [[11], [12], [13]].

In addition to increased technical rigor, attention grew over the ensuing decades to focus on greater evaluation and understanding of the appropriateness (to assess overuse) and necessity (to assess underuse) of medical services. A series of landmark interdisciplinary studies conducted at the RAND Corporation in the 1980s and 1990s highlighted clinical variation in practice and advocated for more explicit criteria to guide diagnosis and treatment [[14], [15], [16], [17]]. These and other studies served as the basis for more rigorous assessment of clinical evidence, evaluation of practice against that evidence, examination of individual care practices against recognized best practices, and ultimately structuring of payment models to align reimbursement incentives with appropriateness [18].

Over the past 4 decades, the discipline of medical quality assessment and improvement has evolved, both scientifically and culturally. Quality control became quality assessment and improvement, and quality improvement became performance improvement. What we recognized originally as performance improvement took a major shift at the turn of the century when the Institute of Medicine published their landmark book *To Err Is Human: Building a Safer Health System* [19]. This publication and the subsequent publication *Crossing the Quality Chasm: A New Health System for the 21*st *Century*, refocused the dialogue from quality to patient safety [20]. The study and improvement of quality has seen many names over the years; recognizing the goal as patient safety focused attention on what matters most to patients, their safety when seeking care from healthcare systems that exist to reduce the burden of disease and improve outcomes of care [21].

Patient safety continues to be a primary focus both within healthcare institutions and clinical laboratories. Five decades ago, the laboratory medicine community recognized that laboratory quality depended not only on the analytic quality of the laboratory, but also on the steps that lead up to testing and those that follow. Laboratory quality experts, including the Centers for Disease Control and Prevention, coined the phrase the “Total Testing Process” that encompasses all aspects of testing from the pre-analytical phase starting with formulation of the clinical question that a laboratory test seeks to answer through the post-analytical phase where the result is reported, acted upon, and impacts patient outcomes [[22], [23], [24]]. Studies suggest that the majority (65%–70%) of laboratory errors occur in the preanalytical phase, before the specimen ever reaches the laboratory. Another 15%–20% occur in the postanalytical phase. The fewest errors actually occur in the analytic phase of the total testing process, that part of the total testing process most under the laboratory's direct control [25].

## 2 Emergence of laboratory stewardship

Laboratory stewardship programs focus primarily on aspects of laboratory practice that offer the greatest opportunity to improve patient care, particularly including the pre- and post-analytical components of the total testing process [26]. As such, they now receive increased attention in the overall approach to optimize laboratory services. Stewardship programs challenge laboratory professionals to reach outside their analytic “comfort zone,” recognizing that the greatest opportunity to impact the value of laboratory testing comes from engaging external colleagues to focus on pre- and post-analytic components [27]. Accordingly, laboratorians are strongly encouraged to further develop their existing relationships with clinical partners by engaging in conversations on what constitutes optimal patient care for achieving desired clinical outcomes and reduced diagnostic errors [6].

The holistic approach of laboratory stewardship to enhance laboratory value aligns with broad healthcare aims. Over the past few decades, healthcare leaders articulated and refined a “triple aim” that includes these key goals: 1) better patient experience; 2) better population health; and 3) lower cost [28]. An additional aim, the well-being of the care team was later added [29]. Laboratory stewardship supports each of these goals: optimal laboratory testing that avoids over- or undertesting improves the patient experience; reduced care variation improves population health; prevention of unnecessary test orders (and associated downstream care) lowers healthcare costs; and streamlined clinical decision support improves clinician practice satisfaction. In addition to these healthcare goals, laboratory stewardship also acknowledges the operational aspects of laboratory medicine, including risk management and reasonable payment from third party payers for medically necessary services [30].

Opportunities to improve laboratory services exist in every organization. Some of these opportunities may be unique to a specific organization, while many are common across multiple healthcare systems. When challenges to quality patient care are common, specialty societies, payers, consortia of health systems, and others will articulate those issues and promulgate guidelines, recommendations, payment policies and other programs to close these common gaps. When developed by these healthcare stakeholders, strategies for optimal care are usually specific, explicit, and based on published evidence. Published guidelines and recommendations from trusted organizations are a good starting place for examination of internal practices because they are defensible, recognized by internal stakeholders, and frequently already tied to performance incentives. Many measures are either laboratory medicine–specific or include laboratory diagnostics metrics to measure performance (eg, percent of patients with diabetes who are regularly evaluated for glucose control).

The Healthcare Effectiveness Data and Information Set (HEDIS®) and the Choosing Wisely® campaign are examples of integrated efforts to identify measures that improve quality of patient care, including laboratory services. HEDIS began in the 1990s and is now one of the most widely adopted tools for improving organizational performance. This set of metrics is developed by the National Committee for Quality Assurance (NCQA) and is used by health plans and medical groups to evaluate clinician performance against accepted metrics [31]. Provider incentives are frequently tied to performance on accepted HEDIS measures.

The Choosing Wisely campaign was initiated by the American Board of Internal Medicine (ABIM) Foundation in 2012 to promote conversations among physicians, patients, and other healthcare stakeholders on appropriate patient care [32]. In 2013, the American Society for Clinical Pathology (ASCP) joined Choosing Wisely to start conversation on the appropriate utilization of medical tests and procedures that sometimes provide little or no benefit and, in some cases, cause harm to patients. A multiyear initiative, the Choosing Wisely campaign provides resources for patients and physicians to engage in these important conversations that help patients choose care that is supported by evidence, not duplicative of other tests and procedures already received, free from harm, and truly appropriate. Choosing Wisely pulls together recommendations from many specialty societies based on published, peer-reviewed evidence. These recommendations, in addition to those published separately by medical specialty societies, are good sources to use when identifying or justifying specific performance improvement projects supported by laboratory stewardship initiatives.

National medical societies such as the ASCP that joined the Choosing Wisely campaign initially identified 5 tests or procedures often used in their specialty whose use, at least in some situations, should be reconsidered. Numerous Choosing Wisely guidelines that focus on laboratory medicine topics now exist, being submitted by laboratory societies (eg, ASCP, the American Association of Blood Banks, the American Society for Clinical Laboratory Science, the American Society for Microbiology) and by specialty societies that use laboratory tests (eg, American College of Obstetricians and Gynecologists, American Academy of Family Physicians) [33,34]. For example, in August 2019 the American Academy of Pediatrics recommended not testing for Lyme disease as a cause of musculoskeletal symptoms without an exposure history or appropriate exam findings. They noted: “The musculoskeletal manifestations of Lyme disease include brief attacks of arthralgia with early disseminated Lyme and/or intermittent or persistent episodes of arthritis in one or a few large joints, with predilection for the knee, in late disease. Lyme testing in the absence of these features and without appropriate exposure from living in or traveling to a Lyme endemic area increases the likelihood of false positive results and may lead to unnecessary follow-up and therapy. Diffuse arthralgias, myalgias, or fibromyalgia alone are not criteria for musculoskeletal Lyme disease.” [35]

## 3 Implementation of a laboratory stewardship program

Successfully implementing a laboratory stewardship program requires 5 key elements: a clear vision and organizational alignment, skills, resources, incentives, and a plan of action. Each element, with examples of implementation, is described in detail below.

### 3.1 A clear vision and organizational alignment

Implementing a laboratory stewardship program is an opportunity for laboratory professionals to drive the change they want to see. It is important, however, that the laboratory's vision (ie, aspirations) is aligned with that of the organization. Absent a clear vision and organizational support there will be confusion with little chance for meaningful success. Successful program implementation may require a change or alignment of culture (ie, the values, beliefs, and norms influencing how people behave as members of an organization) and a shared understanding of why change is needed [36]. Critical to this change management is a solid laboratory professional–clinician partnership.

#### 3.1.1 Understand the health system organization and establish key partnerships with stakeholders

When initiating a laboratory stewardship program, one must first understand the health system's organization and culture. Laboratory stewardship is by nature interdisciplinary, and understanding the health system enables identification of key stakeholders across the organization with whom laboratory professionals will need to partner to achieve desired clinical outcomes. Key stakeholders often include health system medical (eg, physicians, nurses, physician assistants, pharmacists) and administrative (eg, safety managers, information technology analysts, quality improvement specialists, members of the “C-suite”) leadership in addition to laboratory medical and administrative leadership.

Traditional organizational structures are changing in response to pressures throughout healthcare; now, organizational structures vary such that an understanding of one does not likely extend to an understanding of others. Traditional departmental medical staff structures still maintain important roles, but alternative structures such as service or product lines have developed to provide an interdisciplinary, integrated care approach when managing specific patient populations (eg, cardiology, oncology). Therefore, understand how physicians, other healthcare providers, and administrators interact to achieve desired system outcomes so that the stewardship initiative can be integrated smoothly. For example, leaders and departments tasked with improving budget and financial scorecards may have specific targets; laboratory initiatives can help them reach their targets. Health systems usually have working committees focused on quality, infection control, medication management, and medical staff privileging. An existing committee may already be tasked with elements of laboratory stewardship, in which case the stewardship initiative should be integrated carefully and with buy-in from these stakeholders.

Given the importance of physician leadership to every laboratory stewardship program, time is needed to understand the medical staff who use laboratory services. Some physicians may be employed; others might be independent or contracted. These relationships may become relevant when selecting program participants. Alignment may be more difficult when physicians are independent because if their income is generated solely through direct patient care, investing many hours on an “extracurricular” endeavor results in lost income, unless the system compensates individuals for time lost from direct patient care.

#### 3.1.2 Establish leadership and governance for the laboratory stewardship program

A laboratory stewardship program requires dedicated oversight to focus on identifying and implementing needed changes. Before establishing the program's oversight structure, however, recognize how the key administrative (eg, CEO/COO for the system, or the chief technologist in the laboratory) and medical (eg, CMO/director of medical affairs for the system, or laboratory director/CLIA certificate holder in the laboratory) leaders intersect in terms of their roles and responsibilities. This is important both to make sure the right people are engaged as initiatives are undertaken and to assure that organizational structures and lines of authority are respected.

**Fig. 1:**
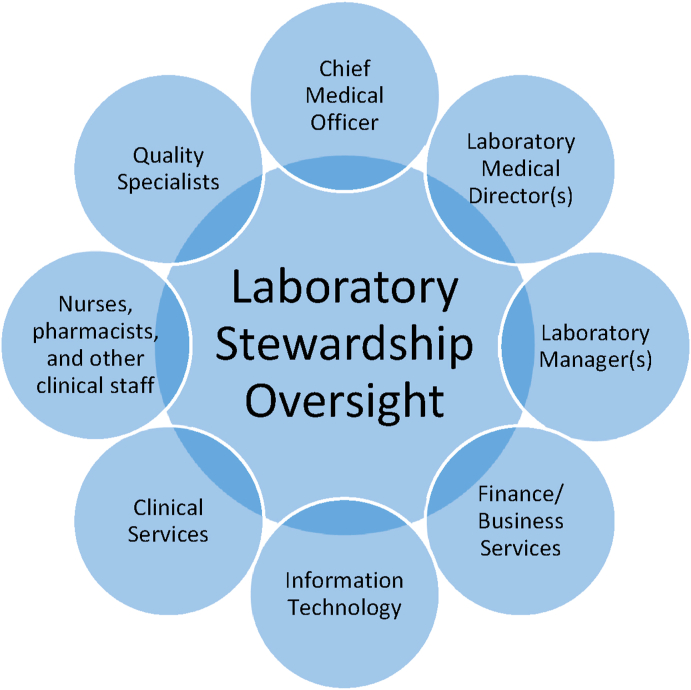
**Possible structure for a laboratory stewardship oversight committee.** Regardless of the oversight structure established in a particular health system, participants in laboratory stewardship oversight should include stakeholders from the laboratory, clinic, and health system administration.

The oversight structure may be a specific committee or a subcommittee or task force of an existing health system committee (Fig. 1). Participants in stewardship oversight should include stakeholders from the laboratory, inpatient and ambulatory providers, and administrative leaders [26]. Laboratory participants may include pathologists, operational directors, and medical laboratory scientists. Clinical participants may include primary care and specialist clinicians and other staff (eg, nurse practitioners, nurses, and physician assistants). Health system administrators (eg, finance, information technology, risk management and process improvement) are important to help integrate and align the program with the broader organization. Additional individuals may participate based on their subject matter expertise related to specific program initiatives.

Successful programs have a specific point of accountability (eg, a designated leader or two co-leaders). There must also be a knowledgeable staff person (eg, program manager) specifically assigned to the laboratory stewardship initiative. The staff person must keep the program on track by aggregating data, coordinating analysts and other team member activities, scheduling meetings, engaging subject matter experts, and ensuring the leader or co-leaders are always apprised of activities.

#### 3.1.3 Communicate a compelling case for change

Stakeholders must be convinced of the need for a laboratory stewardship program. Laboratory-based stewardship champions must create enthusiasm to secure non-laboratory stakeholder buy-in to create the true interdisciplinary team. Once non-laboratory stakeholders support the program, they can also become stewardship champions who help others recognize the value of laboratory services and secure additional buy-in for the program. The interdisciplinary approach helps assure initial efforts are integrated, make operational sense, and progress toward achieving desired clinical outcomes.

Laboratory professionals can actively cultivate other stakeholders to become stewardship champions. Laboratory medical and technologist members who serve on organizational committees may already have established relationships and credibility with patient-facing physicians and clinical staff. Look to existing successful laboratory-hospital partnerships such as effective blood utilization and antimicrobial stewardship initiatives. Laboratory stewardship can thus be framed as an extension of existing partnerships.

Compelling case discussions should include presentation of data (eg, declining performance on HEDIS indicators) to establish the importance of selected initiatives and provide the basis for change, followed by discussions on desired outcomes and how to achieve them. Peer-to-peer conversations, rather than lectures that come across as patronizing, can effectively educate stakeholders and promote alignment. When aligning on desired outcomes, the focus should be on achieving beneficial patient outcomes and not simply achieving appropriate laboratory utilization (eg, correct test ordering patterns). This framing is more inclusive, should align with the organization's strategic plan, and highlights a shared value of laboratorians and clinicians.

Discussions allow stakeholders to share diverse perspectives. Ongoing engagement, especially across departments and disciplines, leads to identification of challenges and opportunities that may not otherwise be recognized. Through collaborative discussion, stewardship champions and key stakeholders can begin developing initial project proposals. Initial projects should each have a reasonable scope so that early program initiatives have the best chance to succeed and prove value. Early successes promote further change and should be widely shared across the organization to build program support. The team can then move on to bigger challenges, including looking at changing deeply engrained practice challenges.

### 3.2 Skills

A laboratory stewardship program requires analytic, subject matter, presentation, project management, interpersonal and leadership skills. Rarely will all the needed skills to carry out a successful laboratory stewardship program be embodied in one person. That is precisely why an effective team brings multiple colleagues to the table, each with unique skills and abilities, to define, evaluate, and address problems.

#### 3.2.1 Analytic skills

Every program must have individuals with the right analytic skills to turn large amounts of laboratory and related institutional data into actionable information. Effective analysts are more than computer programmers or database managers, although the ability to access needed datasets is obviously important. Rather, effective analysts have the ability to turn reams of data from hospital and laboratory information systems into actionable information. The analyst does not necessarily need to have all the clinical knowledge to “massage” the data but must have sufficient grounding to understand what he or she is seeing.

Usually, a preliminary analysis generates more questions than answers. The analyst must have sufficient skills to drill deeper into the data, extract information in different ways, and summarize findings accurately and appropriately. Once familiar with the project and the data, a skilled analyst should ask challenging questions of the entire team. Relevant questions include:

• What are the questions you are seeking to answer? How do they relate to the desired clinical outcomes?
• What clinical and demographic parameters are appropriate?
• Is it appropriate to include all patients, or should the analysis be restricted in some way (eg., outpatients, children under 18, patients with a particular insurance, those with a specific diagnosis or from a particular clinic)?
• Which laboratory tests should be included? Are there any related tests that should be pulled to check the integrity of the data?
• Do we need to know the responsible healthcare providers or their department(s)?
• What time period should we examine?
• Do we need a comparison group by which to judge the results of an intervention? What should be the basis for comparison (eg, using the prior period as a baseline or a comparison group that does not receive the intervention)?
• Is this project entirely focused on internal performance improvement or might it be considered research? (This is an important question because generally projects to improve quality do not require institutional review board [IRB] approval. However, there are exceptions, and it is important to engage an IRB when necessary [37]. Check with your local leadership to make sure you remain in compliance.)

Depending on project complexity and the nature of the data, summarizing the data may best be done in a simple table, a graph, or some type of chart. Many software programs exist that can transform data into easy-to-interpret formats. Regardless of the format, the analyst must be able to share with team members what they did to transform the data. Absent an in-depth team understanding, there is a very high risk that some analyses that look attractive are based on erroneous assumptions, sending the team in the wrong direction or creating an embarrassing situation when presenting findings outside the team.

#### 3.2.2 Subject matter experts

It almost goes without saying that subject matter expertise is an important “skill” that must be represented. In the context of a laboratory stewardship program, subject matter expertise includes both laboratory expertise (eg, laboratory operations, test methodology) and clinical expertise (eg, cardiology, neurology, rheumatology). The required clinical expertise will change based on the specific project or study. Clinical experts serve several roles – so make sure to select the right experts for success. First, clinicians (eg, medical, nursing, pharmacy) will help ground the issues to be addressed and provide feedback during discussions; feedback from this perspective is especially important when the laboratory team wanders too far from clinically relevant issues. Second, when the right clinical leaders are part of and support the initiative, they will be able to positively influence their clinical colleagues to embrace findings and initiate change.

#### 3.2.3 Presentation skills

The best information will have little impact unless it is effectively communicated to stakeholders, whether they are team members, organizational leaders, or scientific colleagues. Know the audience to whom the presentation is directed. An identical presentation given to the team, the health system CEO, and an external group of interested colleagues (eg, at a professional society) will inevitably be either overly general such that it lacks substance, or will contain content that is inappropriate for audiences for whom it was not originally drafted. Each presentation should have a defined goal, acknowledge the needs of the intended audience, and then present data and conclusions aligned with that goal.

Providing detailed instructions on how to give effective presentations is beyond the scope of this discussion. However, publications on audience engagement identify a few key skills that are worth consideration. Garon describes 4 steps leading to a successful presentation [38]:

• Tailor the presentation to your audience (as mentioned above)
• Organize the presentation (introduction, agenda, goals, methods, findings, conclusions, next steps, as appropriate) so that the audience knows where you are headed
• Master presentation techniques by outlining the presentation and practicing it for clarity and timing (eg, present to colleagues or the stewardship committee before presenting outside the group); and
• Use effective visual aids.

A slide deck or handouts can help the audience follow the discussion. When preparing a slide deck, make sure the title of each slide clearly reflects the body of the slide and conveys the message you want the audience to receive. For example, title a slide that presents data about the relationship between chest pain and serum LDL as “Most patients with chest pain have a history of elevated LDL” rather than labeling the slide “Data.” This allows the audience to quickly grasp the take-home message so that they listen to the presenter and do not get lost in the details of the body of the slide. Keep slides brief, graphs simple, and messages clear. Engage with the audience, encourage interaction and questions, and seek their input about the findings that you present.

#### 3.2.4 Leadership, interpersonal, and project management skills

The individual within an organization tasked with leading the laboratory stewardship program must not only know laboratory operations, but they must have the skills, personality, and drive to engage others and make them a part of and understand their value to the project. The individual must have the respect of existing colleagues and the ability to gain the respect of new people. They must sell laboratory stewardship outside the laboratory, have confidence in the data, and be sufficiently visionary to see where an effective program will lead.

Leaders must convincingly convey information to others in ways that result in both buy-in and co-ownership of the findings and resultant action plans. Convincing communication requires trust among colleagues, a well-thought-out synthesis of findings, and a servant leadership philosophy (ie, one that values interaction and recognizes that everybody wins when participants feel empowered). Even the most motivated team members will face anxiety if they perceive the team to lack the necessary skills and cannot envision a pathway to reach the goal. Laboratory stewardship leaders and staff are therefore well-served by project management skills that include developing reasonable goals and effective project tracking to gauge and communicate progress. The most effective laboratory stewardship leaders often emerge as members of the team with the skills to guide the team, ensure goals are accomplished, and celebrate with the team when success is realized.

### 3.3 Resources

Any successful program, and laboratory stewardship is no exception, must have the necessary resources to successfully realize its vision. Without adequate resources, team members will sense frustration rather than success. Decisions will need to be made on whether to develop and allocate in-house resources or outsource selected components of the program; however, program leadership cannot be outsourced.

#### 3.3.1 Human resources

A stewardship program requires personnel dedicated toward the initiative. At minimum, dedicated time for participation is needed from respective key stakeholders: medical leadership, the laboratory, hospitals and clinics, and administration. The importance of a committed program manager has already been discussed. Depending on the goals and structure of the program, the stewardship team may also require dedicated project-specific manager(s), information technology staff/support, data analysts, financial analysts, educators, and other specialists. There may be a need to supplement internal team member skills with specific external expertise in some areas. Irrespective of who is involved in the effort, individuals must be afforded the time and support required to ensure a successful outcome. Specialty societies (e.g., ASCP [39]) and other organizations (e.g., American Society for Quality [40]) provide educational resources to help interested laboratory professionals acquire skills needed to lead quality initiatives such as laboratory stewardship.

#### 3.3.2 Equipment, supplies, and informational resources

Material equipment and supplies and informational resources are required to support a laboratory stewardship program. Typical equipment and supplies include software, educational materials, administrative office supplies, and items for team celebration and reward (eg, small gifts, food). Certain supplies, such as software, are often purchased as a subscription or as a service and require sustained financial resources. Other resources, such as IT infrastructure, may be shared with others in the organization to reduce cost and complexity.

Access to reliable informational resources provides guidance so that program objectives are in accordance with current best practices. The stewardship team should have access to relevant professional societies and their published clinical guidance, including statements and evidence-based guidelines. The Choosing Wisely initiative mentioned previously is a helpful resource for clinical guidance that is publicly available. The laboratory recommendations collected from participating medical societies pertain to 5 categories of test utilization that may inform program objectives: high-cost and unreimbursed testing, repeat normal testing, repetitive testing, obsolete or unproven tests, and clinical pathway diversions. Other helpful informational resources may include payer policies. Because third-party payers incorporate peer-reviewed publications into payment policies, examining those policies is often useful for obtaining current guidance. In general payers link their coverage policies to their payment policies, so this information can also provide insight regarding denials for services provided.

### 3.4 Incentives

#### 3.4.1 Incentives for health systems

Healthcare systems, hospitals of all types, and patient practices all articulate organizational missions and visions. A mission statement drives the organization by providing objectives and strategies to achieve those objectives. The vision statement is forward-looking and identifies where an organization seeks to be in the future. Given the central role of laboratory medicine in every clinical practice, an effective laboratory stewardship program helps any organization reach its goals and objectives, supporting the mission and realizing the vision.

Achieving a mission and vision requires an organization to focus on key aspects related to their practice or operation, including the quadruple aim previously discussed [29]:

• Improving patient experience with care
• Improving the health of populations, including a focus on health equity
• Reduction in the cost of healthcare
• Improving provider satisfaction with care

The following sections discuss how effective integration of laboratory stewardship efforts enhance each of these aims, creating a powerful incentive for any system and its members to engage with the program.

##### 3.4.1.1 Improving patient experience with care

A positive patient experience with care depends on the health system achieving expected quality goals, reflected by a safe experience, expected outcomes, and satisfaction with care. Health systems are incentivized financially by health plans and government payers to improve and maintain quality patient experiences. Within their communities, health systems leverage results of positive patient experience assessments (eg, HEDIS scores, US News and World Report Rankings, recognition in local media) to raise the recognition of their systems as centers of excellence for patient care.

Patient experience depends on the efficient functioning of all parts of the health system. While patient-facing laboratory operations (eg, positive phlebotomy experience, timely and accurate results, accurate invoicing) are important areas of focus for improving patient experience, the information gained through analyses of laboratory performance and utilization informs improvement across systems by guiding appropriate care management, eliminating redundancies, and identifying opportunities to reduce overuse and underuse of medical services.

Effective test utilization, timely result reporting, and a positive patient experience are all metrics that must be included in a comprehensive laboratory stewardship program. When integrated with other health system metrics, laboratory stewardship will be a significant contributor to optimizing patient experience with care.

##### 3.4.1.2 Improving the health of populations

The last decade has focused increasingly on closing gaps in care, particularly among people of color, individuals with limited resources, and those who are challenged with respect to health literacy. Addressing the health of populations requires attention to more than health services. Health systems committed to population health must be sensitive to all social determinants of health dimensions because many non-healthcare delivery factors have a bigger overall impact on health than the direct provision of healthcare services (Table 1).

**Table 1:** The social determinants of health [41].

Social and Economic Factors (40%)	Health Behaviors (30%)
•Employment •Income •Education level •Family and social support •Community safety	•Diet and exercise •Alcohol/substance use •Sexual activity •Smoking
**Physical Environment (10%)**	**Clinical Care (20%)**
•Air and water quality •Housing and transportation	•Access to care •Quality of care

Improving population health requires patients to have access to health services, comfort seeking those services, and knowledge about which services to receive and when. Guideline-driven early screening and preventive services are a key component to improving population healthcare. Beyond impacting the 20% of health driven by healthcare, data and information provided by laboratory stewardship initiatives, with appropriate integration, offer the ability to impact health behaviors and social and economic factors that represent the largest contributions to population health. For example, analysis of laboratory data may reveal missing screening services or new abnormal laboratory test results that may, when probed, indicate resource limitations (eg, job layoff, loss of safe living conditions) that cause modification of eating habits, substance use, or access to medication.

##### 3.4.1.3 Reducing healthcare costs

Laboratory stewardship may achieve its initial traction in a health system by providing insights to reduce test overutilization. Overuse is ubiquitous in both the inpatient and ambulatory settings and takes multiple forms. In the inpatient setting patients may receive daily testing for stable conditions. This type of overuse often results from standing orders or the employment of order sets where those ordering the laboratory services select a set of tests rather than selecting each test individually. That is not to say that order sets are inappropriate — they can guide appropriate test selection, but it is important to medically validate the specific content of the order set. In addition, tests ordered during an inpatient hospital encounter may be adversely impacted by acute disease, producing misleading results; they are instead best ordered after the patient is discharged, during a future ambulatory visit.

In the ambulatory setting, laboratory services are vulnerable to overuse and underuse, particularly when care is not delivered as part of an integrated delivery network. For example, overuse occurs when there is repeated ordering of the same tests by multiple providers or when the ordering of tests is more frequent than specified by clinical guidelines for monitoring chronic conditions. Information management may be challenging if an individual laboratory does not have insight into all test orders and results (eg, one clinician orders a test performed by the health system laboratory and another orders the same test from a reference laboratory whose results are not integrated with the system).

Addressing the cost of care also must consider downstream consequences of laboratory diagnostics, both when patients receive services they do not need and when they are not offered necessary services. Underuse of relatively inexpensive laboratory diagnostics often dramatically raises long-term costs of care because the resultant delays in screening may increase adverse consequences of disease, such as more advanced cancer, poorer diabetes control, and unaddressed dangerous lipid profiles. These issues were highlighted in 2020 during the COVID-19 pandemic where different patient characteristics resulted in different risk levels for disease and disease severity [42]. During the pandemic, the biggest patient threat has likely been underutilization, particularly among tests to manage chronic diseases and in traditionally underserved communities and people of color [43,44]. Laboratory professionals have access to data that can provide insight into clinical practices and identify potentially pandemic-related gaps in care. A laboratory stewardship program provides a venue for systematic data analysis and positions laboratory professionals as healthcare leaders with a commitment to appropriate, equitable, and efficient care [43].

##### 3.4.1.4 Improving provider satisfaction

This discussion does not specifically focus on the fourth healthcare aim; however, optimized laboratory resources, operations, and staff assure clinicians receive the right test, for the right patient, at the right time, ultimately having a positive influence on provider satisfaction with their profession.

#### 3.4.2 Incentives for laboratory stewardship team members and participants

At the outset, and throughout the course of a program initiative, it is important to keep the team motivated. Motivation requires that team members, team leaders, and the organization feel positive about selected initiatives and receive internal and external support to continue their engagement. As an organization launches or continues its laboratory stewardship journey, leaders must assure that the team members maintain the motivation and dedication that initially brought them to the table. This requires ongoing incentives for members that align with their values and motivation.

Many people assume that the most powerful incentives are financial; however, money may be but one of numerous incentives that effectively drive a laboratory stewardship team toward success. Laboratory professionals (including pathologists) are just that, professionals. As described below, team members may feel incentivized by increased recognition and satisfaction, the ability to contribute to improved patient care, and the opportunity for career advancement. Whatever the incentives, they must be relevant to those engaged in the stewardship program. Without incentives there may be tacit acceptance of the program, but progress, at best, will be slow as other initiatives receive higher priority.

##### 3.4.2.1 Gratitude, recognition, and celebrations of success

Regular acts of gratitude and recognition from organizational leadership and peers encourage initial engagement and ongoing involvement with a stewardship program. Recognizing and celebrating team success boosts morale and helps link the program's success with a more positive and communal workplace culture, addressing a fundamental and universal human need to be acknowledged by others and for efforts to be valued.

Employee appreciation and recognition programs have become common in the workplace. Psychological research supports their importance; expressions of gratitude lead to enhanced job performance and prosocial behavior [45]. In healthcare settings, studies demonstrate that a positive workplace culture, including effective teamwork and change management, reduces clinician burnout [46]. These findings are salient because about 44% of physicians in the US reportedly experience symptoms of burnout, as measured by emotional exhaustion and depersonalization scales [47]. If the workforce responsible for executing prescribed changes exhibits burnout and low morale, then implementing changes in practice areas, such as how these practitioners use laboratory services, will encounter more challenges and resistance (eg, “not another organization initiative!“). Acts of gratitude, recognition, and celebration thus create a positive feedback loop: these acts foster a more positive workplace culture that increases the willingness to change, and a positive culture with effective change management helps reduce burnout, leading to improved performance, higher morale, and more occasions for acts of gratitude, recognition, and celebration.

##### 3.4.2.2 Tangible expressions of gratitude

Although the above discussion focuses on primarily intangible expressions, it is important not to dismiss the value of tangible expressions of appreciation. Financial recognition is neither nefarious nor inappropriate, and certainly some team members may appreciate this type of recognition as much as or more than the team celebrations. This may particularly be the case during times of financial crisis when healthcare professionals struggle to provide for their families.

Successful laboratory stewardship programs usually improve operational efficiencies and often have both direct and indirect financial returns. Sharing some of those returns with the laboratory stewardship team, even to a small extent, sends the message that the organization supports and appreciates the efforts and is committed to sharing gains that result from improvement initiatives. The specifics of tangible sharing of success clearly depend on the system where the improvement occurs. Increased financial remuneration may be direct through a team “bonus”, through promotion to a higher-level position with an increased salary range, or through positive performance reviews in those systems that link salary to performance evaluations.

##### 3.4.2.3 Improved patient care

The mission of a health system focuses on providing high-quality patient care. Physicians, laboratorians, and other healthcare providers who provide this care commit to ethical medical practice. The healthcare professionals who will lead and participate in laboratory stewardship programs are thus imbued with an ethical imperative to provide healthcare services that adhere to best practices in patient care, including effective test utilization [48,49].

Inappropriate test ordering is inconsistent with quality patient care and goes against the ethical principle of beneficence (ie, acting in the best interest of the patient). Examples of patient harm from improper laboratory testing include false-positive results (and unnecessary follow-up care), additional patient discomfort, and increased cost. Laboratory stewardship seeks to provide the right test to the right patient at the right time. This alignment provides an innate incentive for engagement when healthcare professionals view the laboratory stewardship program as an effective vehicle for employing ethical principles.

This innate incentive relies on intrinsic rather than extrinsic motivation. That is, an innate incentive to engage in change initiatives such as laboratory stewardship comes from characteristics of the work itself rather than external factors such as financial rewards. Evidence from physician surveys and quality improvement collaboratives suggests that clinicians indeed act upon intrinsic motivation to improve patient outcomes [50]. Laboratory stewardship programs would be well-served to design and frame initiatives such that they harness this intrinsic motivation that incentivizes engagement and participation.

##### 3.4.2.4 Accelerated career development

Opportunity for accelerated career development can be an additional incentive for engaging with a laboratory stewardship program. Provided that the program has sufficient structure with well-defined objectives and metrics, participants and teams can note concrete progress and achievements. As mentioned above, team members benefit from recognition of achievements during celebrations of success, but achievements may be leveraged beyond short-term celebrations to influence long-term career trajectory.

Organizational leadership can create incentives related to career development by recognizing and rewarding high-achieving individuals and teams with beneficial opportunities. Opportunities may include appointments to committees that allow individuals to spearhead future organizational initiatives. Participants who are already in leadership positions can be rewarded with increased resources for their projects, such as increased staff and upgraded equipment. Those who are involved in research activities can be rewarded with publication support and resources for presenting at prominent conferences. Opportunities such as these build influence and prestige for individuals and, in turn, the health system.

### 3.5 A plan of action

An effective laboratory stewardship program requires dedication, planning, and clear direction with a clear statement articulating the need for change and a declaration of the problem to be solved. As change management authority John Kotter notes in his book *Leading Change*, one of the first steps is “creating a sense of urgency.” [51] Among healthcare professionals, including laboratory professionals, this urgency is best cultivated by simple, effective messaging condensed to *what is the why, what is the problem to be solved,* and ultimately *what's in it for me* (see previous discussion on incentives).

A well-defined plan of action includes this simple messaging and articulates program objectives, with milestones and metrics. The plan facilitates effective communication, assures a set of common goals, and drives progress. When progress is judged against a plan, it becomes easier to identify best practices and obstacles or barriers that warrant a midcourse adjustment.

The plan should incorporate the four other elements discussed previously — organizational vision and alignment, skills, resources, and incentives. To succeed, the laboratory stewardship team and organizational management must agree on and execute according to the plan. Without clear direction, even the best intentions will be met with false starts and wasted time.

#### 3.5.1 Define the problems, identify desired outcomes, and align goals

Using the team's analytical skills and capabilities, the laboratory stewardship program leaders begin to plan initiatives by defining specific challenges to address and obtaining accurate baseline data from which improvement can be judged. The team can then articulate, using the same metrics, the desired outcome(s) and develop a plan to move from the baseline to the goal.

The action plan must include securing the required resources (eg, time, funding, staffing support) to ensure identified goals are attainable. An action plan must also provide for consistent engagement with stakeholders and focus on health system priorities by selecting projects that are clearly defined and achievable, consistent with available resources.

#### 3.5.2 Plan specific interventions and establish an ongoing process improvement discipline

After defining the problems to address and the desired clinical outcomes, the laboratory stewardship committee plans specific interventions to achieve the program's goals. The committee needs to decide what interventions to implement, how to implement them, and when changes will take effect. There are many kinds of interventions that the committee may consider, with stringency ranging from gentle to strong in their ability to curtail ineffective test utilization [26]. Gentle interventions are typically educational and do not involve systematic changes (eg, posting guidelines or incorporating electronic reminders). Medium-strength interventions involve systematic changes but allow navigation around them (eg, hiding tests in an electronic ordering system but allowing them to be ordered if specifically requested). Strong interventions involve systematic changes with hard stops to prevent certain behaviors (eg, alerts in an electronic ordering system that a test cannot be ordered with the specified criteria).

Throughout the laboratory stewardship program, process improvement should be an ongoing discipline and incorporated into team culture. A common model used for process improvement is the FOCUS-PDCA cycle: Find a process improvement opportunity, Organize a team with relevant knowledge, Clarify current knowledge of the process, Understand the problem(s) with the process, and Select the action to be taken for process improvement. FOCUS is followed by PDCA: Plan the improvements, Do the improvements, Check the results of these actions, and Act to further implement changes (if they are successful) or stop and adjust (if they are not successful). PDCA can be repeated as a cycle while a particular opportunity is pursued. The PDCA approach ensures regular check-ins on interventions and provides opportunities to raise issues or bring ideas for improvement [52]. Regular check-ins also ensure the program's goals remain aligned to those of the health system.

## 4 Putting it all into practice: a hospital-based case study^1^

Huntington Health System is a large, 800-bed, 3-hospital system in a metropolitan midwestern region that includes 700,000 residents. The system has 17 ambulatory practices, including 4 ambulatory surgery centers, throughout its service area. Approximately 65% of local residents have used Huntington Health in the previous 3 years. Over half of the physicians treating Huntington patients, while independent practitioners, practice solely at Huntington facilities; the remainder are in community practice and at times admit patients to the hospitals. Huntington's flagship hospital is a 450-bed facility that contains the system's main clinical laboratory. The other hospitals and clinics offer urgent laboratory services but refer most routine and advanced testing to the main laboratory. The main laboratory performs most testing requested by their providers; they refer 3% of requested tests to reference laboratories that serve their community.

Huntington Health System is preparing for their upcoming accreditation survey. The laboratory manager attends a planning meeting where the system leadership team inquires whether any departments have performance improvement activities that they would like to showcase during the upcoming survey. The laboratory manager reminds the team that the clinical laboratory implemented a laboratory stewardship program 18 months ago and recently completed their College of American Pathologists (CAP) Laboratory Accreditation semi-annual inspection. The laboratory was particularly proud of their stewardship activities and the engagement of the entire system in response to findings that emerged from the program. In fact, CAP surveyors specifically complimented on the program when reviewing laboratory standard GEN.20316 (QM indicators of quality): “The QM program includes monitoring key indicators of quality in the pre-analytic, analytic, and post-analytic phases by regularly comparing performance against targets defined by the laboratory.” [53] The inspector commented that the program was particularly responsive to the following key quality indicator specified in the standards: “Laboratory Test Utilization: Percent of tests (or a test) that appear to be redundant, excessive or noncontributory to good patient care.” The laboratory manager agrees to work with the laboratory director to summarize their laboratory stewardship journey as an example of an interdisciplinary Huntington program that improved patient care while simultaneously reducing costs.

The setting: About 2 years ago, the laboratory manager attended a conference that presented strategies for laboratory stewardship. Soon afterwards, she assembled a small group of laboratorians, including the laboratory medical director, the head of the clinical chemistry and microbiology sections, and the lead technologists in each of those sections, for an initial discussion on laboratory stewardship opportunities at Huntington. She also invited to the initial discussion Huntington's associate director for ancillary services, to whom the laboratory reports. The laboratory manager presented some initial data she retrieved from the laboratory information system regarding test ordering practices along with laboratory-specific claims denial data (from their ambulatory practices) for commonly ordered tests.

Based on their review, this initial team determined that they wanted to better understand laboratory testing trends for their patients, particularly around sexually transmitted illnesses, HIV testing, hepatitis C testing, and thyroid testing. The data suggested that there may be opportunities within these higher volume services to eliminate duplicate orders and standardize ordering patterns. The next steps were to 1) normalize the data so that all Huntington Health System facilities could be aggregated together for an “apples to apples” comparison; 2) select 1 or more specific tests for improvement; and 3) present the plan to Huntington's Performance Improvement Committee to attain leadership's endorsement and identify others to be part of the team.

The laboratory manager met with the laboratory information systems support team and defined the critical data elements they required, the study period, and the timeline for the project. They requested the data elements below for a 6-month period (Table 2). Because the initial discussions focused on ambulatory patients, they restricted the analysis to patients seen in their ambulatory settings and excluded patients seen in the emergency department, inpatient, or hospital observation settings. There was a brief discussion regarding whether Huntington intended to publish the findings; however, the team decided this was an internal quality improvement project and there was no intent to publish at the time, so an Institutional Review Board exemption was not required.

**Table 2:** Example of data requested for initial analysis of laboratory usage.

Medical Record Number	Accession Number	Collection Date/Time	Patient Age	Diagnosis codes (ICD-10)	Test Name	Test Result	Result Flags	Ordering provider	Ordering Location

The laboratory team analyzed the data and saw considerable variation in ordering practices across all the tests they examined. They decided to focus initially on thyroid testing and pulled the following Choosing Wisely recommendation as a guide: “Don't order multiple tests in the initial evaluation of a patient with suspected thyroid disease. Order thyroid-stimulating hormone (TSH), and if abnormal, follow up with additional evaluation or treatment depending on the findings.”

The manager, working with the system billing department, identified several key payer policies that addressed appropriateness of thyroid testing. They retrieved Medicare's National Coverage Determination number 190.22 for Thyroid Testing along with the diagnosis codes that Medicare indicated were appropriate when testing was ordered. They also reviewed coverage policies for United Healthcare, Aetna, Cigna, Humana, and Blue Cross/Blue Shield.

The team focused their study more specifically on the following CPT®/HCPCS codes: 84436 and 84439 (Thyroxine [T4], total and free, respectively); 84480–84482 (Triiodothyronine [T3], total, free, and reverse, respectively); and 84443 (Thyroid Stimulating Hormone). Based on the Choosing Wisely guidelines, they restricted their analysis to patients being screened for thyroid disease – coded with International Classification of Disease (ICD-10) code Z13.29 (Encounter for Screening for other Suspected Endocrine Disorder). By definition, appropriate application of this screening code would preclude patients known to have a thyroid disorder because tests for patients with thyroid disease are considered diagnostic services, not screening services. The screening code was listed (without an accompanying thyroid disease code that would suggest the test intention was diagnostic) in just under half the patients for whom any of the above thyroid tests were ordered.

The laboratory quality and performance improvement specialist developed a dashboard that showed testing volumes by location that included situations where 1 or more thyroid tests were ordered. These dashboards quickly showed variation in ordering patterns by physicians within a particular practice and differences between practices. More screening tests were ordered by certain primary care practices (eg, general internal medicine, family medicine, obstetrics) compared to specialty practices (eg, endocrinology, endocrine surgery). Two primary challenges were identified: 1) duplicate testing within a short period of time; and 2) ordering of multiple thyroid tests for patients receiving initial screening for symptoms. Analysis also showed that many requests for thyroid testing were not accompanied by an appropriate ICD-10 code to support the medical necessity of the test(s). The laboratory director brought the initial findings to Huntington's next Performance Improvement Committee meeting and both physician members and hospital administration provided positive feedback and encouraged the laboratory to move forward.

Armed with these findings, the project team expanded to include an endocrinologist, a family physician, and a geriatrician (general internist). After reviewing detailed data, there was less concern about specialist test ordering, given the nature of their practices. However, review of medical records showed that 3 endocrinologists would use ICD-10 screening code Z13.29 for the patients they were managing with known thyroid disease. The endocrinologist on the team agreed to reach out to the colleagues who were misapplying the screening code and educate them about when to report a screening service versus a diagnostic service.

Requesting multiple thyroid tests for initial evaluation of patients with appropriate symptoms was, as noted, more common among the generalists. The laboratory director, endocrinologist, and internist together developed an educational brochure that conveyed: 1) the appropriate indications for thyroid screening, 2) the United States Preventive Services Task Force recommendations related to screening [54], and 3) a recommended Huntington Health System guideline for appropriate thyroid testing. Detailed data analysis identified that 10% of the generalist medical staff ordered 60% of multi-test thyroid panels for initial assessment. Two practices, representing 8% of the generalist medical staff, were the most frequent outliers. The endocrinologist and family physician reached out to those practice medical directors and discovered that these practices historically had an order set that included multiple thyroid tests and it was this order set, rather than TSH, that was being selected when patients were seen. The practices agreed to change their ordering menu to promote effective test utilization.

**Fig. 2:**
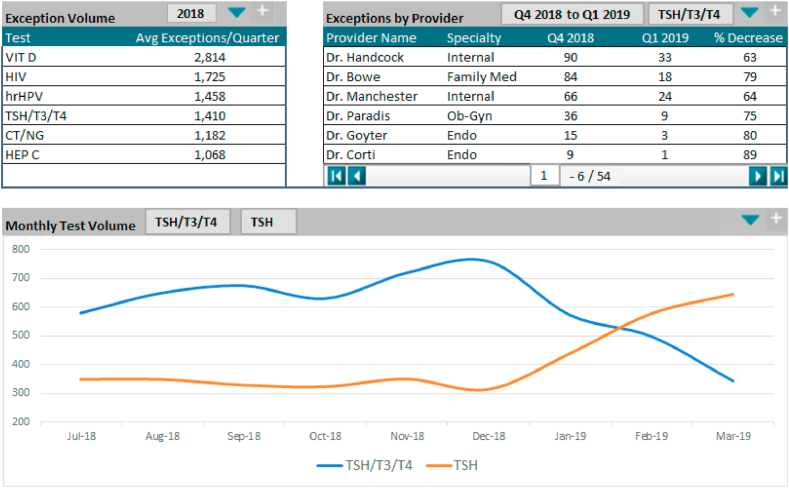
**Laboratory Stewardship Data Dashboard for Thyroid Screening Orders, First Quarter 2019.** The upper left box shows exceptions to the health system's test ordering guidelines for commonly selected tests. The upper right box shows clinician-specific impact of the thyroid testing intervention when the system promulgated the rules (showing only the providers with the highest number of exceptions). The bottom chart shows migration from the inappropriate screening and adoption of the appropriately-indicated TSH test for initial screening. CT, *Chlamydia trachomatis*; Endo, endocrinology; HEP C, hepatitis C; HIV, human immunodeficiency virus; hrHPV, high-risk human papillomavirus; NG, *Neisseria gonorrhoeae*; Ob-Gyn, obstetrics and gynecology; T3, triiodothyronine; T4, thyroxine; TSH, thyroid-stimulating hormone; VIT D, vitamin D.

Four months after initiating the project, the laboratory pulled ordering practices for the first full quarter following their initial meeting (Fig. 2). Generalist panel testing for initial encounters for suspected thyroid disease decreased almost 70% from the baseline data. Specialist physician use of ICD-10 Z13.29 became rare as those physicians began assigning the correct diagnosis codes when seeing patients with known thyroid disease. The system finance department reported that third-party payer denials declined by 30%.

The team reexamined the data and determined, as previously noted, that many claims were submitted without a supporting ICD-10 code (eg, those identified as appropriate by Medicare). They discovered this was a billing issue and not a practice issue. Specifically, given the complexity of Huntington patients, over half of their ambulatory patients had claims with 8 or more diagnoses. The laboratory processing system, however, reported only the first 4 ICD-10 codes on their claims. These 4 ICD-10 codes more frequently reflected the diagnostic reasons for the patient visit to the physician and not the reason for the thyroid test. Therefore, the appropriate ICD-10 codes related to indications for thyroid testing were included in the original claim, but in positions that did not reach the final claim. The finance department edited their billing system to expand the number of ICD-10 codes submitted with claims so that all relevant diagnoses were reflected on the submission. This change resulted in considerably fewer denials.

The clinical laboratory reported these findings to the Performance Improvement Committee. The committee was impressed with the laboratory stewardship initiative, the interdisciplinary collaboration between the laboratory and the clinical staff, and, of course, the improvement in effective test utilization. The laboratory manager invited the laboratory team and key Huntington leadership to a celebration to recognize the stewardship achievement. The CEO came to the recognition and announced that the “Thyroid Testing Improvement Team” would receive a recognition bonus the following month, as a token of appreciation.

Using everything the team learned from this laboratory stewardship project (data analytics, interdisciplinary relationships, performance improvement approaches), they agreed to keep going. Their next initiatives will focus on effective test utilization for the diagnosis and management of diabetes mellitus and chronic kidney disease. The laboratory team assured Huntington that not only will they be happy to share this initiative at the upcoming accreditation survey, but they will have projects to showcase for future surveys as well.

## 5 Conclusion

Laboratory medicine is central to the clinical practice of medicine, touching more patients than any other medical discipline. Health systems often take their laboratories for granted because the results are almost always timely and accurate. However, the laboratory itself may be somewhat siloed from the clinical and other departments (eg, pharmacy or imaging) in some systems where the laboratory team stays focused on their internal operations and lacks regular direct contact with ordering physicians and other clinicians. This separation may lead to misalignment on appropriate laboratory ordering practices or misinterpretation of test results. As a result, quality of patient care can be adversely impacted and misutilization of laboratory testing unnecessarily increases the cost of care. Avoiding these outcomes by aligning the laboratory with all stakeholders across the health system thus benefits both the system and patients.

Appropriate laboratory testing is therefore critical in today's healthcare environment that aims to improve patient care while reducing cost. Laboratory stewardship presents a valuable opportunity for laboratory professionals to engage with clinical colleagues and drive change toward realizing these aims. Implementing an effective laboratory utilization program builds upon principles of change management. Central to the program's success are the engagement and partnership with clinical and administrative colleagues who support implementation of program initiatives. Using clinical data as the basis for change, laboratory professionals can cultivate organizational support for proactively improving laboratory use. In so doing, laboratory professionals take a leading role in providing high-quality patient care and exercise leadership within their health systems.

## Declaration of competing interest

Terra White and Lee Hilborne are employed by Quest Diagnostics. Lee Hilborne owns Quest Diagnostics stock. Diane Janowiak is employed by hc1. Wesley Wong is a consultant for hc1 and receives compensation for his services.

This research did not receive any specific grant from funding agencies in the public, commercial, or not-for-profit sectors.

## Footnotes

1 The health system, personnel, and scenario described below are fictitious and are intended as an illustrative example of the principles discussed. The discussion is based, however, on an actual scenario.
